# Factors predicting amoxicillin prescribing in primary care among children: a cohort study

**DOI:** 10.3399/BJGP.2021.0639

**Published:** 2022-07-12

**Authors:** Faith Miller, Ania Zylbersztejn, Graziella Favarato, Imad Adamestam, Lucy Pembrey, Laura Shallcross, Dan Mason, John Wright, Pia Hardelid

**Affiliations:** Institute for Global Health, University College London, London.; Great Ormond Street Institute of Child Health, University College London, London.; Great Ormond Street Institute of Child Health, University College London, London.; College of Medicine and Veterinary Medicine, The University of Edinburgh, Edinburgh.; Department of Medical Statistics, London School of Hygiene and Tropical Medicine, London.; Institute of Health Informatics, University College London, London.; Bradford Institute for Health Research, Bradford.; Bradford Institute for Health Research, Bradford.; Great Ormond Street Institute of Child Health, University College London, London.

**Keywords:** anti-bacterial agents, drug prescriptions, drug resistance, medical record linkage, paediatrics, respiratory tract infections

## Abstract

**Background:**

Antibiotic prescribing during childhood, most commonly for respiratory tract infections (RTIs), contributes to antimicrobial resistance, which is a major public health concern.

**Aim:**

To identify factors associated with amoxicillin prescribing and RTI consultation attendance in young children in primary care.

**Design and setting:**

Cohort study in Bradford spanning pregnancy to age 24 months, collected 2007–2013, linked to electronic primary care and air pollution data.

**Method:**

Amoxicillin prescribing and RTI consultation rates/1000 child–years were calculated. Mixed-effects logistic regression models were fitted with general practice (GP) surgery as the random effect.

**Results:**

The amoxicillin prescribing rate among 2493 children was 710/1000 child–years during year 1 (95% confidence interval [CI] = 677 to 744) and 780/1000 (95% CI = 745 to 816) during year 2. During year 1, odds of amoxicillin prescribing were higher for boys (adjusted odds ratio [aOR] 1.36, 95% CI = 1.14 to 1.61), infants from socioeconomically deprived households (aOR 1.36, 95% CI = 1.00 to 1.86), and infants with a Pakistani ethnic background (with mothers born in the UK [aOR 1.44, 95% CI = 1.06 to 1.94] and outside [aOR 1.42, 95% CI = 1.07 to 1.90]). During year 2, odds of amoxicillin prescribing were higher for infants with a Pakistani ethnic background (with mothers born in the UK [aOR 1.46, 95% CI = 1.10 to 1.94] and outside [aOR 1.56, 95% CI = 1.19 to 2.04]) and those born <39 weeks gestation (aOR 1.20, 95% CI = 1.00 to 1.45). Additional risk factors included caesarean delivery, congenital anomalies, overcrowding, birth season, and childcare attendance, with GP surgery explaining 7%–9% of variation.

**Conclusion:**

Socioeconomic status and ethnic background were associated with amoxicillin prescribing during childhood. Efforts to reduce RTI spread in household and childcare settings may reduce antibiotic prescribing in primary care.

## INTRODUCTION

Antimicrobial resistance is a public health emergency, to which prescribing of antibiotics contributes.^1^ In the UK most antibiotics are prescribed in primary care.^2^^,^^3^ Several studies have found that patient-level factors including gender, flu vaccination status, obesity, smoking, previous antibiotic receipt, and comorbidities increase antibiotic prescribing among adults.^4^^,^^5^ Prescribing rates also vary geographically according to area-level deprivation and the proportion of patients with comorbidities.^6^^,^^7^

Few studies have examined antibiotic use among children, despite a third of children aged <5 years being prescribed at least one antibiotic annually.^3^ Childhood antibiotic prescribing in the UK peaks between the ages of 1 and 4 years, and three-quarters of antibiotics prescribed to children in primary care are for respiratory tract infections (RTIs).^8^^,^^9^ Furthermore, antibiotic prescribing in childhood is linked with adverse health outcomes in later childhood, including asthma and obesity.^10^^,^^11^

The severity of presenting illness determines the likelihood of being prescribed an antibiotic for common childhood infections.^9^ Therefore, the authors expect antibiotic prescribing rates among children to differ according to the factors that increase the risk of contracting common infections, including overcrowding and having older siblings,^12^^,^^13^ and factors that reduce the immune system’s ability to fight infections if exposed, such as prematurity, exposure to air pollution, and lack of breastfeeding.^14^^–^16 Furthermore, qualitative studies suggest parent–clinician communication may have an impact on childhood antibiotic prescribing.^17^ However, few studies have examined risk factors for antibiotic prescribing among children.

This study set out to establish child, family, and environmental factors that were associated with prescribing of amoxicillin to children aged <2 years, to inform antimicrobial stewardship efforts among children. In order to examine the importance of individual and primary care-level factors, the proportion of total variation in amoxicillin prescribing attributed to GP surgery-level variation was calculated.

This study focused on amoxicillin because it is the most commonly prescribed antibiotic in UK primary care.^8^^,^^9^ As amoxicillin is most commonly prescribed for RTIs, the risk factors associated with primary care consultation rates for RTIs were also examined.

## METHOD

### Data sources

Data from the Allergy and Infection (ALL-IN) substudy of the Born in Bradford (BiB) cohort was used in this study. ALL-IN and BiB have been described in detail elsewhere.^18^^,^^19^ Briefly, pregnant women attending the Bradford Royal Infirmary for an oral glucose tolerance test (offered to all women booked for delivery at the Bradford Royal Infirmary at 26–28 weeks of pregnancy) between March 2007 and November 2010 were invited to participate in BiB. Parents completed baseline questionnaires, and details about the birth were extracted from hospital electronic maternity records. Details of congenital anomalies were obtained from a local register linked to the cohort.^20^ Linkage to routinely collected electronic primary care data facilitated follow-up.^18^

**Table:** How this fits in

Prescribing of antibiotics during childhood contributes to antimicrobial resistance, which is a major public health concern. This study linked rich cohort data to routinely collected primary care data to identify ethnic and socioeconomic inequalities in childhood respiratory infections and amoxicillin prescribing. The study highlights that population-level interventions, including reducing household overcrowding and supporting hygiene measures in childcare settings, are required to reduce the need for antibiotic prescribing in young children, thereby supporting antimicrobial stewardship efforts in primary care.

Children born from March 2008 whose parents had completed the BiB baseline questionnaires were invited to participate in the ALL-IN subcohort when aged 11 months.^19^ Families who agreed to participate in ALL-IN completed questionnaires at ages 12 and 24 months (completed February 2009 to September 2013). The study used openly available data on annual average levels of fine particulate matter (≤2.5 µm, PM_2.5_) modelled at a 1 × 1 km grid across the UK using atmospheric transport models, from the Department for Environment, Food and Rural Affairs. PM_2.5_ levels were mapped to lower super output areas (LSOA, a small geographical area with an average population of 1500 people) and linked to the cohort via LSOA of the infant’s home address.^21^^–^23

### Outcome variables

The primary outcome was the receipt of at least one amoxicillin prescription in primary care during the first and second year of life (see Supplementary Table S1).^23^ The secondary outcomes were at least one primary care consultation for upper RTIs (URTIs) or lower RTIs (LRTIs). Diagnostic information from electronic primary care records were recorded using Read codes version 3 (CTV3; see Supplementary Table S2).^24^^–^26

### Exposure variables

Table 1 summarises the source and characteristics of all outcome and exposure variables. Gestational age was coded as a binary variable for being born preterm/early-term (<39 weeks) or term (≥39 weeks). Initially 37 weeks was used to define a variable for preterm/term, however, the number of infants born <37 weeks was low (*n* = 112/2493, 4.5%). The five-category variable defining socioeconomic status had previously been derived from the BiB baseline questionnaire.^27^ Information on whether the child had spent any time in formal childcare (for example, at a nursery or with a childminder), lived in a household with ≥6 people, or had exposure to indoor household pollutants (such as mould/damp or gas cookers) was collected via parental questionnaires.

**Table 1.** Source and characteristics of each variable included in the analysis

**Variable**	**Source**	**Type**	**Coding**
**Primary outcome**			
GP amoxicillin prescribing	Electronic primary care records	Binary	≥1 prescription each year: yes/no

**Secondary outcome**			
GP consultation for URTI	Electronic primary care records	Binary	≥1 consultation each year: yes/no
GP consultation for LRTI	Electronic primary care records	Binary	≥1 consultation each year: yes/no

**Exposure**			
Sex of infant	Maternity records	Binary	Male
			Female
Delivery mode	Maternity records	Binary	Vaginal
			Caesarean
Quarter of birth	Maternity records	Categorical	January–March
			April–June
			July–September
			October–December
Gestational age	Maternity records	Binary	<39 weeks
			≥39 weeks
Congenital anomalies	Congenital anomaly register at Bradford Royal Infirmary	Binary	0 congenital anomalies
			≥1 congenital anomaly
Ethnic background	BiB baseline questionnaire	Categorical	White British
			Pakistani, UK-born
			Pakistan, non-UK born
			Other
Socioeconomic status	BiB baseline questionnaire	Categorical	Least deprived and most educated
			Employed not materially deprived
			Employed with no access to money
			On benefits but coping
			Most deprived
Maternal smoking during pregnancy	BiB baseline questionnaire	Binary	Smoked during pregnancy: yes/no
Breastfeeding duration	ALL-IN 12-month questionnaire	Categorical	<1 month
			1–<6 months
			≥6 months
Childcare	ALL-IN 12-month and 24-month questionnaires	Binary	Child in formal childcare: yes/no
Number of people in household	ALL-IN 12-month and 24-month questionnaires	Binary	Child in overcrowded (6 people) dwelling: yes/no
Household mould/damp	ALL-IN 12-month and 24-month questionnaires	Binary	Child in dwelling with visible mould/damp: yes/no
Gas cooking	ALL-IN 12-month and 24-month questionnaires	Categorical	Gas cooking only
			Gas and electric cooking
			Electric cooking only
Quartile of PM_2.5_ in relation to Bradford level	Department for Environment, Food and	Categorical	1st/2nd quartile
	Rural Affairs linked with LSOA from BiB		3rd quartile
	baseline questionnaire and ALL-IN		4th quartile
	12-month questionnaire		

*ALL-IN = allergy and infection. BiB = Born in Bradford. LRTI = lower respiratory tract infection. LSOA = lower super output area. PM_2.5_ = particulate matter ≤2.5 µm. URTI = upper respiratory tract infection.*

The 1 × 1 km annual ambient PM_2.5_ levels were mapped to the LSOA of the child’s residence at birth and age 1 year. PM_2.5_ exposure during pregnancy was calculated using the average PM_2.5_ level in the calendar year(s) covering the gestation period, weighted by the number of days’ gestation during each calendar year. PM_2.5_ exposure during the first year of life was also weighted by the number of days spanning each calendar year. The authors calculated annual quartiles of average PM_2.5_ levels across the Bradford postcode area and derived two variables to indicate weighted PM_2.5_ exposures at the child’s LSOA, relative to the distribution of PM_2.5_ exposures in the Bradford area, throughout gestation and the first year of life. As few children lived in lower pollution areas, a three-category variable was created to indicate PM_2.5_ exposure during pregnancy and the first year of life: first/second quartile, third quartile, and fourth quartile of BD postcode area levels. Summary statistics for the absolute levels of PM_2.5_ exposure corresponding to each group can be found in Supplementary Table S3.

### Statistical analyses

Amoxicillin prescribing rates, and rates of consultations for URTIs and LRTIs per 1000 child–years for the first and second years of life, overall and according to each exposure variable of interest were calculated. The 95% confidence intervals (CIs) were calculated to compare rates across exposures. Crude and mutually adjusted mixed-effects logistic regression models (‘xtlogit’ function in Stata) were fitted to determine factors associated with receiving at least one amoxicillin prescription in the first and second years of life. GP surgery was included as the random intercept to determine surgery- and child-level variation in prescribing. For the small number of children who changed GP surgery during the year, they were assigned to the surgery they were registered with for longest.

Based on a priori knowledge, all mutually adjusted models included ethnic background, socioeconomic status, and sex as core confounders. Due to the exploratory nature of this study, additional exposure variables were selected for inclusion if they improved model fit, determined using likelihood ratio tests (*P*-value <0.05). Likelihood ratio tests were conducted until no further variables improved model fit. Variables were dropped from the final model if they no longer improved fit in the fully adjusted model. Model fitting and selection procedures were repeated for having at least one URTI or LRTI consultation during the first and second years of life.

In total, 491 participants did not attend the ALL-IN 24-month follow-up. Therefore, multiple imputation using chained (MICE; ‘mi’ function in Stata; 30 imputed datasets) was used to impute data for the 24-month follow-up.^28^ The MICE model included all variables in the substantive model (including outcome variables), variables from the 12-month questionnaires, additional variables predictive of missingness, and GP surgery. To avoid perfect prediction, in which categorical outcomes are predicted almost perfectly resulting in instability during estimation, all GP surgeries with <10 participants were combined into one group (*n* = 68/2493, 2.7%). Models for the second year of life were undertaken with and without MICE as a sensitivity analysis. All analyses were undertaken in Stata (version 16.1).

## RESULTS

The cohort included 2493 singleton children. In total, 2002 (80.3%) attended the 24-month follow-up (see Supplementary Figure S1). In total, 48.9% of all mothers had a Pakistani ethnic background (*n* = 1220/2493) and 43.7% were from the two most deprived socioeconomic groups (*n* = 1089/2493) (Table 2).

**Table 2.** Summary of cohort characteristics, and amoxicillin prescribing rates^a^

**Baseline characteristic**	**Cohort, *n* /*N* (%), (*N*= 2493)**	**Amoxicillin prescribing rate/1000 child–years, rate (95% CI)**	

		**Year 1**	**Year 2**
**Total**		710 (677 to 744)	780 (745 to 816)

**Sex of infant**			
Male	1264/2481 (50.9)	776 (728 to 827)	823 (774 to 875)
Female	1217/2481 (49.1)	642 (597 to 689)	735 (688 to 786)
Missing	12/2493 (0.5)	—	—

**Mother’s ethnic background**			
White British	920/2491 (36.9)	529 (483 to 579)	638 (587 to 692)
Pakistani, UK born	438/2491 (17.6)	845 (760 to 936)	900 (813 to 994)
Pakistani, not UK born	782/2491 (31.4)	969 (901 to 1042)	935 (868 to 1006)
Other	351/2491 (14.1)	436 (369 to 512)	659 (576 to 751)
Missing	2/2493 (0.1)	—	—

**Socioeconomic status**			
Least deprived and most educated	507/2483 (20.4)	608 (541 to 680)	704 (632 to 782)
Employed not materially deprived	443/2491 (17.8)	505 (440 to 576)	711 (632 to 795)
Employed with no access to money	446/2485 (17.9)	641 (568 to 720)	786 (705 to 874)
On benefits but coping	717/2485 (28.9)	872 (805 to 944)	881 (813 to 953)
Most deprived	372/2485 (15.0)	856 (763 to 956)	776 (688 to 872)
Missing	8/2493 (0.3)	—	—

**Mother smoking during pregnancy**			
No	2147/2491 (86.2)	724 (688 to 761)	796 (758 to 835)
Yes	344/2491 (13.8)	617 (536 to 706)	682 (596 to 777)
Missing	2/2493 (0.1)	—	—

**Congenital anomalies**			
No	2409/2493 (96.6)	702 (669 to 737)	763 (728 to 799)
Yes	84/2493 (3.4)	940 (743 to 1173)	1270 (1039 to 1537)
Missing	0/2493 (0.5)	—	—

**Gestational age**			
Term/late-term	1796/2481 (72.4)	703 (665 to 744)	749 (710 to 791)
Early/preterm	685/2481 (27.6)	729 (666 to 796)	862 (793 to 935)
Missing	12/2493 (0.5)	—	—

**Quarter of birth**			
January–March	643/2493 (25.8)	696 (632 to 764)	731 (665 to 801)
April–June	601/2493 (24.1)	833 (761 to 910)	775 (706 to 850)
July–September	608/2493 (24.4)	694 (629 to 765)	805 (735 to 880)
October–December	641/2493 (25.7)	624 (565 to 690)	810 (741 to 883)
Missing	0/2493 (0.0)	—	—

**Delivery mode**			
Vaginal	1964/2481 (79.2)	693 (656 to 731)	768 (729 to 808)
Caesarean	517/2481 (20.8)	778 (703 to 859)	829 (751 to 912)
Missing	12/2493 (0.5)	—	—

**Breastfeeding duration, months**			
<1	1096/2482 (44.2)	774 (723 to 829)	841 (787 to 898)
1–<6	598/2482 (24.1)	706 (639 to 777)	765 (696 to 839)
≥6	788/2482 (31.7)	622 (567 to 680)	714 (656 to 776)
Missing	11/2493 (0.4)	—	—

**Characteristics collected at 12 months and 24 months**	**Cohort, *n* /*N* % (*N*= 2493)**		**Amoxicillin prescribing rate/1000 child-years, rate (95% CI)**	
	
	**Year 1**	**Year 2**	**Year 1**	**Year 2**
**Quartile of PM_2.5_ in relation to Bradford level^b^**				
1st/2nd quartile	904/2493 (36.3)	839/2476 (33.9)	623 (573 to 678)	802 (742 to 865)
3rd quartile	1116/2493 (44.8)	1039/2476 (42.0)	769 (718 to 822)	784 (731 to 840)
4th quartile	473/2493 (19.0)	598/2476 (24.2)	738 (662 to 821)	750 (681 to 824)
Missing	0/2493 (0.0)	17/2493 (0.7)	—	—

**Formal childcare attendance**				
No	2038/2482 (82.1)	1511/1935 (78.1)	746 (708 to 784)	793 (748 to 839)
Yes	444/2482 (17.9)	424/1935 (21.9)	553 (485 to 627)	852 (766 to 945)
Missing	11/2493 (0.4)	558/2493 (22.4)	—	—

**Household mould/damp**				
No mould or damp	1912/2475 (77.3)	1571/2001 (78.5)	702 (665 to 741)	808 (764 to 854)
Mould or damp	563/2475 (22.7)	430/2001 (21.5)	746 (676 to 822)	803 (720 to 893)
Missing	18/2493 (0.7)	492/2493 (19.7)	—	—

**Number of people in household**				
2–5	1719/2482 (69.3)	1343/1994 (67.4)	593 (557 to 631)	705 (666 to 747)
≥6	763/2482 (30.7)	651/1994 (32.6)	977 (908 to 1050)	948 (880 to 1020)
Missing	11/2493 (0.4)	499/2493 (20.0)	—	—

**Cooking type**				
Electric cooking only	312/2481 (12.6)	226/2002 (11.3)	560 (480 to 651)	635 (534 to 750)
Electric and gas cooking	467/2481 (18.8)	361/2002 (18.0)	569 (502 to 643)	699 (614 to 791)
Gas cooking only	1702/2481 (68.6)	1415/2002 (70.7)	777 (735 to 821)	862 (814 to 912)
Missing	12/2493 (0.5)	491/2493 (19.7)	—	—

a *Amoxicillin prescribing rates per 1000 child–years during the first 2 years of life for the total cohort, and summarised according to exposure categories.*

b *PM_2.5_ exposure for year 1 represents exposure in utero and year 2 represents exposure during the first year. The median and IQR for the absolute PM_2.5_ levels corresponding to each quartile are displayed in Supplementary Table S3. IQR = interquartile range. PM_2.5_ = particulate matter ≤2.5 µm.*

### Amoxicillin prescribing

A total of 1594/2493 children (63.9%) received ≥1 amoxicillin prescription during the first 2 years of life; 42.5% (*n* = 1060/2493) during the first year, and 46.9% (*n* = 1169/2493) during the second year (see Supplementary Table S4). Prescribing rates were 710 (95% CI = 677 to 744) per 1000 child–years during their first year of life, and 780 (95% CI = 745 to 816) during the second year (Table 2). Table 3 displays the best-fitting model for amoxicillin prescribing during the first and second years of life. Compared with children of White British mothers, the odds of receiving at least one amoxicillin prescription during the first year of life were higher for children with mothers with a Pakistani ethnic background, regardless of their country of birth (UK-born Pakistani mothers: adjusted odds ratio [aOR] 1.44, 95% CI = 1.06 to 1.94; Pakistani mothers born outside UK: aOR 1.42, 95% CI = 1.07 to 1.90) and lower for those with ‘other’ ethnic backgrounds (aOR 0.70, 95% CI = 0.52 to 0.96). Infants from the most deprived households had higher odds of being prescribed amoxicillin (aOR 1.36, 95% CI = 1.00 to 1.86) compared with the least deprived.

**Table 3.** Associations between exposures and amoxicillin prescribing during the first 2 years of life^a^

**Variable**	**Amoxicillin prescribing, OR (95% CI)**			

	**Year 1**		**Year 2^b^**	

	**Crude**	**Mutually adjusted (*n*= 2450)**	**Crude**	**Mutually adjusted (*n*= 2476)**
**Sex of infant**				
Male	1.33 (1.13 to 1.58)	1.36 (1.14 to 1.61)	1.13 (0.96 to 1.33)	1.14 (0.96 to 1.34)
Female	1.00 (ref)	1.00 (ref)	1.00 (ref)	1.00 (ref)

**Mother’s ethnic background**				
White British	1.00 (ref)	1.00 (ref)	1.00 (ref)	1.00 (ref)
Pakistani, UK born	1.44 (1.09 to 1.89)	1.44 (1.06 to 1.94)	1.36 (1.04 to 1.77)	1.46 (1.10 to 1.94)
Pakistani, not UK born	1.48 (1.16 to 1.91)	1.42 (1.07 to 1.90)	1.40 (1.10 to 1.77)	1.56 (1.19 to 2.04)
Other	0.68 (0.51 to 0.90)	0.70 (0.52 to 0.96)	0.88 (0.67 to 1.15)	0.98 (0.74 to 1.31)

**Socioeconomic status**				
Least deprived and most educated	1.00 (ref)	1.00 (ref)	1.00 (ref)	1.00 (ref)
Employed not materially deprived	0.82 (0.62 to 1.09)	0.79 (0.59 to 1.06)	1.15 (0.88 to 1.50)	1.13 (0.85 to 1.50)
Employed with no access to money	0.95 (0.72 to 1.25)	0.92 (0.69 to 1.22)	1.14 (0.88 to 1.50)	1.11 (0.85 to 1.46)
On benefits but coping	1.13 (0.88 to 1.45)	0.92 (0.70 to 1.21)	1.38 (1.08 to 1.76)	1.26 (0.97 to 1.64)
Most deprived	1.41 (1.06 to 1.87)	1.36 (1.00 to 1.86)	1.28 (0.96 to 1.69)	1.26 (0.93 to 1.70)

**Mother smoking during pregnancy**				
No	1.00 (ref)	—	1.00 (ref)	—
Yes	1.02 (0.80 to 1.32)	—	1.00 (0.78 to 1.27)	—

**Congenital anomalies**				
No	1.00 (ref)	1.00 (ref)	1.00 (ref)	1.00 (ref)
Yes	1.78 (1.12 to 2.83)	1.63 (1.01 to 2.63)	1.78 (1.11 to 2.83)	1.57 (0.98 to 2.51)

**Gestational age**				
Term/late-term	1.00 (ref)	1.00 (ref)	1.00 (ref)	1.00 (ref)
Early/preterm	0.99 (0.82 to 1.20)	0.97 (0.80 to 1.18)	1.22 (1.01 to 1.46)	1.20 (1.00 to 1.45)

**Quarter of birth**				
January–March	1.00 (ref)	1.00 (ref)	1.00 (ref)	—
April–June	1.26 (0.99 to 1.60)	1.33 (1.04 to 1.69)	1.13 (0.89 to 1.42)	—
July–September	0.99 (0.78 to 1.26)	0.99 (0.77 to 1.26)	1.20 (0.95 to 1.51)	—
October–December	0.94 (0.74 to 1.18)	0.91 (0.71 to 1.15)	1.27 (1.01 to 1.59)	—

**Delivery mode**				
Vaginal	1.00 (ref)	1.00 (ref)	1.00 (ref)	1.00 (ref)
Caesarean	1.21 (0.99 to 1.49)	1.23 (1.00 to 1.53)	1.10 (0.90 to 1.35)	1.08 (0.88 to 1.32)

**Breastfeeding duration, months**				
<1	1.29 (1.05 to 1.57)	1.21 (0.97 to 1.50)	1.29 (1.07 to 1.57)	1.22 (0.99 to 1.50)
1–<6	1.17 (0.93 to 1.47)	1.11 (0.87 to 1.41)	1.06 (0.85 to 1.33)	0.98 (0.78 to 1.24)
≥6	1.00 (ref)	1.00 (ref)	1.00 (ref)	1.00 (ref)

**Quartile of PM_2.5_ in relation to Bradford level^c^**				
1st/2nd quartile	1.00 (ref)	—	1.00 (ref)	1.00 (ref)
3rd quartile	1.11 (0.91 to 1.37)	—	0.99 (0.81 to 1.23)	0.94 (0.76 to 1.16)
4th quartile	1.03 (0.78 to 1.36)	—	1.03 (0.80 to 1.32)	0.97 (0.75 to 1.26)

**Formal childcare**				
No	1.00 (ref)	1.00 (ref)	1.00 (ref)	1.00 (ref)
Yes	0.99 (0.79 to 1.25)	1.29 (1.00 to 1.66)	1.22 (0.97 to 1.53)	1.45 (1.12 to 1.87)

**Household mould/damp**				
No mould or damp	1.00 (ref)	1.00 (ref)	1.00 (ref)	—
Mould or damp	1.01 (0.82 to 1.23)	0.98 (0.80 to 1.21)	0.98 (0.79 to 1.21)	—

**Number of people in household**				
2–5	1.00 (ref)	1.00 (ref)	1.00 (ref)	—
≥6	1.54 (1.27 to 1.86)	1.41 (1.14 to 1.74)	1.23 (1.02 to 1.49)	—

**Cooking type**				
Electric cooking only	1.00 (ref)	—	1.00 (ref)	—
Electric and gas cooking	1.14 (0.84 to 1.56)	—	1.18 (0.84 to 1.66)	—
Gas cooking only	1.17 (0.90 to 1.54)	—	1.40 (1.05 to 1.86)	—

**Intra-GP surgery correlation coefficient**	—	0.09 (0.06 to 0.15)	—	0.07 (0.04 to 0.11)

a *Crude and mutually adjusted models for amoxicillin prescribing during the first and second years of life. All models included GP surgery as the mixed-effect. Mutually adjusted models were adjusted for the mother’s ethnic background, socioeconomic status, and infant sex a priori, as well as: (a) first year of life: congenital anomalies, gestational age, quarter of birth, delivery mode, breastfeeding duration, formal childcare attendance, household mould or damp, and household overcrowding; (b) second year of life: congenital anomalies, gestational age, delivery mode, breastfeeding duration, PM_2.5_* *exposure, and formal childcare attendance.*

b *Models for year 2 include variables imputed using multivariate imputation.*

c *The median and IQR for the absolute PM_2.5_ levels corresponding to each quartile are displayed in Supplementary Table S3. OR = odds ratio. PM_2.5_ = particulate matter 2.5 µm. ref = reference.*

Odds were also higher for infants who were male, had at least one congenital anomaly, born in April–June (compared with January–March), born via caesarean section, living in a household with ≥6 people, and attending formal childcare. Based on the intraclass correlation coefficient, GP surgery-level variation explained 9% (95% CI = 6 to 15) of the residual variation in amoxicillin prescribing during year 1.

The odds of receiving at least one amoxicillin prescription during the second year of life were higher for children with mothers with a Pakistani ethnic background (regardless of whether mothers were born within [aOR 1.46, 95% CI = 1.10 to 1.94] or outside of [aOR 1.56, 95% CI = 1.19 to 2.04] the UK) compared with children with White British mothers. Children born preterm/early-term and those attending formal childcare also had higher odds of amoxicillin prescribing (aOR 1.20, 95% CI = 1.00 to 1.45 and 1.45, 95% CI = 1.12 to 1.87, respectively). GP surgery-level variation explained 7% (95% CI = 4 to 11) of the residual variation in amoxicillin prescribing during year 2.

No significant associations were observed between amoxicillin prescribing and maternal smoking during pregnancy, breastfeeding duration, nor exposure to environmental PM_2.5_, household mould or damp, or household gas cooking during either year. Model results based on complete cases were comparable (see Supplementary Table S5).

### GP consultations for RTIs

Rates of URTI consultations were 309 (95% CI = 287 to 332) per 1000 child–years during the first year of life and 263 (95% CI = 243 to 284) during the second year. Rates of LRTI consultations were 458 (95% CI = 432 to 486) per 1000 child–years during the first year and 409 (95% CI = 384 to 435) during the second year (see Supplementary Table S6).

Associations were observed between the odds of having at least one URTI GP consultation and ethnic background and breastfeeding duration during years 1 and 2 (see Supplementary Table S7). In total, 13% (95% CI = 8 to 20) of the residual variation in URTI consultations was explained by GP surgery-level variation during year 1 and 14% (95% CI = 9 to 22) during year 2.

For LRTIs, significant associations were observed between GP consultation attendance and sex, ethnicity, and socioeconomic status during year 1, and ethnicity, socioeconomic status, congenital anomalies, delivery mode, and childcare attendance during year 2 (see Supplementary Table S8). In total, 11% of the residual variation in LRTI consultations (95% CI = 7 to 18) during year 1 and 15% (95% CI = 9 to 23) during year 2, was explained by GP surgery-level variation. No significant associations were observed between RTI consultations and maternal smoking, season of birth, gestational age at delivery, household overcrowding, nor exposure to ambient air pollution, household mould or damp, or household gas cooking.

## DISCUSSION

### Summary

This study found that 42.5% (*n* = 1060/2493) of children were prescribed amoxicillin at least once during their first year of life and 46.9% (*n* = 1169/2493) during their second year.

Having a mother with a Pakistani ethnic background, irrespective of their country of birth, and childcare attendance were associated with amoxicillin prescribing across both years. Socioeconomic status, birth characteristics, and household overcrowding were significantly associated with amoxicillin prescribing during the first year only, whereas prematurity was significantly associated with amoxicillin prescribing during the second year only. Less than 10% of the total variance was attributed to GP surgery-level variation in amoxicillin prescribing.

Ethnic background and childcare attendance were significantly associated with having at least one primary care consultation for URTIs and LRTIs. Breastfeeding status was associated with URTI consultation attendance only, whereas socioeconomic status, congenital anomalies, and delivery mode were associated with LRTI consultation attendance only. In total, 11%–15% of the variation in the probability of attending at least one RTI consultation was attributed to GP surgery-level variation.

### Strengths and limitations

A strength of this study is the comprehensive analysis of risk factors for amoxicillin prescribing, enabled by linking rich, longitudinal questionnaire data to routinely collected maternity and primary care records, and air pollution data. The ethnic diversity of the BiB cohort makes it particularly well suited to study ethnic differences in child health outcomes.

However, first, the data include children growing up in Bradford, and a limitation is therefore that this may not be representative of all children in England. Second, some environmental exposures were collected by parental report and may be subject to recall bias. Third, the indications for antibiotic prescribing were not examined, as these are inconsistently recorded in primary care.^29^

Further, this study focused on prescribing in primary care, excluding prescribing for more severe indications in hospitals.^2^ Lastly, the prescribing data were collected between 2008 and 2013, before the development of the UK 5-year antimicrobial resistance strategy to reduce overprescribing of antibiotics.^2^ Despite these limitations, these data are well suited to provide new insights on amoxicillin prescribing patterns among children in England.

### Comparison with existing literature

Rates of amoxicillin prescribing in the current study are considerably higher than previous estimates, which may reflect the characteristics of the BiB cohort (see Supplementary Table S8).^3^^,^^6^ These findings of an increased likelihood of amoxicillin prescribing for more deprived children and children with mothers with a Pakistani ethnic background mirror findings in adults.^4^^,^^5^^,^^19^ Ethnic differences in prescribing may reflect differences in the severity of presenting illness, which is a key determinant for antibiotic prescribing, or differences in population mixing, parental expectation, or clinician response.^30^

Increased odds of amoxicillin prescribing during the first year of life for the most deprived children was observed. Significant socioeconomic differences were also observed in GP consultation attendance for LRTIs but not URTIs, suggesting infection severity differs by socioeconomic background. The socioeconomic disparity may be more significant than the current study’s results suggest, as poorer BiB mothers are less likely to consult primary care once health status is taken into account.^31^ Socioeconomic differences were found to be more profound for LRTI consultations compared with amoxicillin prescribing, which may indicate that antibiotic prescribing guidelines reduce socioeconomic differences in prescribing, but not incidence of infection.^32^^,^^33^

Primary care physicians play a vital role in reducing antibiotic prescribing. A modest but significant variation in amoxicillin prescribing was identified between practices (7%–9%). This is smaller than previous estimates, which range from 32.0% to 65.0%.^6^^,^^34^^,^^35^ However, previous studies have included data from across England, whereas the GP surgeries in this study all reside within the Bradford District and Craven Clinical Commissioning Group. Residual variation in antibiotic prescribing may reflect differences in provider services and prescribing practices, or differences in health need. This study also found higher variation in childhood RTI consultation attendance between GP surgeries, hinting that GP surgery-level variation in prescribing may be driven by differences in health need or individual access to services, rather than the GP surgery’s prescribing practices. Although previous studies suggest that targeting physician behaviour can reduce antibiotic prescribing,^36^ these findings highlight the importance of interventions at the population level. Further research considering detailed individual-level characteristics and indicators of infection risk (including overcrowding and childcare attendance) would determine where population-level antimicrobial stewardship efforts are best targeted.

Surprisingly, associations between maternal smoking or outdoor air pollution and RTI consultation attendance or amoxicillin prescribing were not observed. Regarding maternal smoking, this may be unique to the Bradford population because of the disparity between smoking habits observed between mothers of White British and Pakistani ethnic background. Furthermore, self-report of maternal smoking may not represent true behaviours; research has shown that mothers from less deprived areas are less likely to report their smoking during pregnancy.^37^ PM_2.5_ has previously been found to increase the risk of URTIs and LRTIs.^38^^,^^39^ However, given that small effect sizes are expected, larger studies over broader geographical areas with greater variation in PM_2.5_ exposure are required to address its role in RTIs and antibiotic prescribing.^40^ Linking datasets on environmental exposures to newly available national primary care dispensing data could aid in these studies.^41^

### Implications for research and practice

These findings highlight the need for policies addressing the population-level inequalities associated with RTIs when addressing antimicrobial stewardship efforts among children, particularly ethnic background and socioeconomic status. Primary care networks, through which primary care practices link with other health and social care providers, provide an opportunity for GPs to promote handwashing and improved ventilation in homes (particularly those that are overcrowded) and childcare settings to prevent the spread of infection. Partnerships with pharmacies and voluntary sector organisations, for example, could help facilitate these efforts. Furthermore, this study found that children who were breastfed for <1 month were more likely to attend consultations for URTIs. There are useful published resources to aid GPs when providing breastfeeding support, including those published by the UK GP Infant Feeding Network.^42^^,^^43^ Primary care networks could also play a role in linking primary care to local breastfeeding support groups. Larger, national studies investigating the effect of environmental exposures on childhood respiratory health and antibiotic prescribing are recommended.

## Data

The data that support the findings of this study are available from several different sources, some of which are openly available, others available only on request. Restrictions apply to the availability of data from the BiB and ALL-IN cohorts, which are not publicly available and used under license for the study. Data are available from authors on reasonable request and with permission of BiB. PM_2.5_ data are available at: https://uk-air.defra.gov.uk/data/pcm-data.
